# Medical Items

**Published:** 1912-02

**Authors:** 


### MEDICAL ITEMS

### ABDOMINAL SUPPORT IN PREGNANCY.

The wisdom of supporting the abdomen during the last stages of pregnancy and occasionally from the very beginning, is becoming more generally recognized. The advantages have been conclusively demonstrated, not alone by assuring greater comfort, but quite as substantially by the preventon of many of the disagreeable and more or less serious complications of pregnancy traeable to abdominal sagging. The large amount of thought that has been given to the proposition is shown by the development of special forms of support. Unquestionably any measure or appliance approaching closest to every day customs and requring the least possible change in a patient's usual manner of dress, desedvss special consideration. To the painstaking medical man the Storm Binder is bound to present a special appeal. Careful scientific study of the anatomical requirements are reflected in this splendid maternity supporter, and the physician is bound to commend the effective support afforded without forcing a woman to wear an unnatural and unpleasant apparatus.

The Storm Abdominal Binder solves a most important probliem and the benefits obtained from its use show how perfectly adapted it is to the necessarily exacting needs of the pregnant female. The comfort that attends its use is a feature second only to the complete support it constantly gives. Limited space prevents elaboration of the many important and interesting facts connected with the Storm Binder, and every physician who is interested in promoting the welfare of his pregnant patients should turn to page 4 and send forthwith for full description.

---

Tongaline exerts a manifest action on the nervous system of the secreting order of glands, it diminishes the uric acid content of the blood and produces substitutive irritaton in the region of the articular surfaces. On account of the exaggerated deposits toward the emunctories, causing a great secretion of bile in the liver, an abundant diuresis in the kidneys and a serous diarrhoea in the intestines, while in the faeces and in the urine we find a great quantity of uric acid.

These conditions secure the attainment of the desired effect, which is to expel from the organism all those agents, the accumulation of which in the blood are the cause of rheumatism, neuralgia, grippe, gout, nervous headache, malaria, sciatica, lumbago, tonsillitis, heavy colds and excess of uric acid.

---

### STORM BINDER—THE FAVORITE OF THE MEDICAL PROFESSION.

We note with much pleasure the wonderful growth of the Storm Binder in the favor of the medical profession. From a comparatively small beginning but a few years ago the business has grown into a large and profitable one. Dr. Katherine L. Storm, the inventor and head of the concern, is to be congratulated on this success, which has been won through the worth of her binder and her fair dealing. Dr. Storm not only has the satisfaction of having built up a paying business, but she also has the greater satisfaction of having scores of grateful patients to whom her name is a synonym for relief and comfort. The testimony of the numbers she has helped in various conditions through the efficacy of her excellent binder and supporter means more to Dr. Storm than any other phase of her success. Probably no other binder on the market has to so great a degree the favor and confidence of the medical profession. The Journal rather especially rejoices in the success of this woman physician.

---

For some years there has been a tendency among the profession to use sodium salicylate to the practical exclusion of other agents in the treatment of acute articular rheumatism. Even the therapeutic nihilist has conceded to sodium salicylate the first place in rheumatism, as he accords it to quinine in malaria, or to antitoxin in diphtheria."

"The recent reports by Menzer, a surgeon in the German army, tend to discredit the too implicit faith placed in the use of the salicylates in acute articular rheumatism. It is not denied that the salicylates pushed to effect will alleviate the syymptoms, but he asserts that cases so treated are more subject to relapse and to permanent deformity than are those in which other remedies are exhibited, and that there should be incorporated with the salicylates other agents which will not encourage injurious complications."

In Tongaline, sodium salicylate from the natural oil is combined with tonga, colchicum, black cohosh and pilocarpin, whereby the organs of elimination are greatly stimulated, so that prompt and efficient results are secured without the necessity of such large doses of the salicylates as to cause any harmful effects.

---

### TISSUE NUTRITION IN CONVALESCENCE.

If grip were free from treacherous sequelae, the physician could dismiss his grip patient after the acute period of the disease had passed, feeling sure that an uneventful return to health would soon follow. But these sequelae strike when least expected. The heart muscle fails, with resulting acute dilatation; or a tuberculous taint manifests itself. If it were made a routine practice to insist that grip convalescents take a tissue food of proven merit, such as Cord. Ext. Ol. Morrhuae Comp. (Hagee), the complications and sequelae of this infection would not be met so frequently and in less distressing form. Cord. Ext. Ol. Morrhuae Comp. (Hagee) contains the very elements the drained system needs to restore it to health and vigor, the contained extractives of the cod liver oil, coupled with the hypophosphites of lime and sodium, supplying this need in admirable manner.

